# Investigation of the correlation between diabetic retinopathy and prevalent and incident migraine in a national cohort study

**DOI:** 10.1038/s41598-022-16793-0

**Published:** 2022-07-20

**Authors:** Anna Stage Vergmann, Lonny Stokholm, Katrine Hass Rubin, Anne Thykjær, Sören Möller, Caroline Schmidt Laugesen, Steffen Heegaard, Kurt Højlund, Ryo Kawasaki, Katja Christina Schielke, Jakob Grauslund

**Affiliations:** 1 Department of Ophthalmology, Odense University Hospital, Odense, Denmark; 2 Department of Clinical Research, University of Southern Denmark, Odense, Denmark; 3 OPEN–Open Patient data Explorative Network, Odense University Hospital & University of Southern Denmark, Odense, Denmark; 4 Steno Diabetes Center Odense, Odense University Hospital, Odense, Denmark; 5 Department of Ophthalmology, Zealand University Hospital Roskilde, Roskilde, Denmark; 6 Department of Ophthalmology, Rigshospitalet Glostrup, Copenhagen, Denmark; 7 Department of Clinical Medicine, University of Copenhagen, Copenhagen, Denmark; 8 Department of Vision Informatics, University of Osaka, Osaka, Japan; 9 Department of Ophthalmology, Aalborg University Hospital, Aalborg, Denmark

**Keywords:** Diabetes, Headache

## Abstract

Migraine is a disease characterized by cerebral vasodilation. While diabetes has previously been associated with a lower risk of migraine, it is not known if diabetic retinopathy (DR), a retinal peripheral vascular occlusive disease, is a potential biomarker of protection against migraine. Therefore, we aimed to examine diabetic retinopathy as a marker of prevalent and 5-year incident migraine. In a national cohort, we compared patients with diabetes attending DR screening from The Danish National Registry of Diabetic Retinopathy (cases, n = 205,970) to an age- and gender-matched group of patients without diabetes (controls, n = 1,003,170). In the cross-sectional study, a multivariable model demonstrated a lower prevalence of migraine among cases compared with controls (OR 0.83, 95% CI 0.81–0.85), with a lower risk in cases with DR than in those without (OR 0.69, 95% CI 0.65–0.72). In the prospective study, a lower risk of incident migraine was found in a multivariable model in cases (HR 0.76, 95% CI 0.70–0.82), but this did not depend upon the presence of DR. To conclude, in a national study of more than 1.2 million people, patients screened for DR had a lower risk of present migraine, but DR was not a protective marker of incident migraine.

## Introduction

Migraine is a comprehensive and disabling disease that affects around 11% of the adult population worldwide^1^. It is believed to originate from a combination of environmental, genetic, and hormonal causes, with an overrepresentation in women^2–4^. Diseases such as heart disease and hypertension are associated with an increased the risk of developing migraine^5^. Previous findings have reported a relation between migraine and the occurrence of hypoglycemia attacks and longer duration of type 1 diabetes^6^. However, there are conflicting results in this field as both type 1 and type 2 diabetes have been associated with a decreased risk of migraine, especially in older patients^7–9^. The pathophysiological explanation for this is not entirely clarified, but a proposed mechanism involves both a neurogenic and a vascular component^10^.

It is commonly known that diabetes leads to long term macro- and microvascular complications such as cardiovascular disease (including hypertension), peripheral neuropathy, nephropathy and diabetic retinopathy (DR)^11^. The vascular damage includes endothelial dysfunction and increased angiogenesis that consequently cause arteriosclerosis and reduced blood flow to nerves^12,13^. Migraine is believed to be caused by a combination of vasogenic and neurogenic factors with vasodilation of vessels and overactivation of impulses from sensory nerves^10^. In diabetes and in patients with retinal vascular abnormalities, the damage of nerves and the alteration of vessels from arteriosclerosis could be a plausible explanation of the lower prevalence of migraine in patients with diabetes^14,15^. The level of DR is known to increase in severity with longer duration of disease, poorly controlled HbA1c, increased blood lipids and hypertension^16–20^, making DR a good indicator of disease severity in diabetes. An insight into the association between level of DR and migraine could contribute to a better understanding of the overall impact of DR and the interaction between DR and extraocular diseases.

Therefore, the purpose of this study was to examine DR as a marker of prevalent and 5-year incident migraine in a national cohort of patients, who have attended DR-screening in comparison with an age- and gender-matched group of non-diabetec controls.

## Research design and methods

This study was a matched register-based cohort study. The study population consisted of Danish patients with diabetes registered in The Danish National Registry of Diabetic Retinopathy (DiaBase)^21^. The DiaBase is a national quality database containing data of 205,970 patients with diabetes, who attend the national screening program for DR. Patients are screened by ophthalmologists according to evidence-based national clinical guidelines, and it is mandatory to report screening results to the database. Data includes the level of DR, which is graded according to the International Clinical DR severity scale^22^ as levels 0 (no DR), 1–3 (mild, moderate, and severe non-proliferative DR) or 4 (proliferative DR). The level of DR was determined by the worse eye. Grading of DR was performed as a combination between evaluation by fundus photos and fundus evaluation by slit-lamp bio microscopy or with one of the methods alone. The following registers and databases were used to collect and link data: the Danish National Prescription Registry (DNPR)^23^, National Patient Register (NPR), and the Danish Civil Registration System (CPR)^24^. A further description of each database can be found elsewhere^25^.

Patients with diabetes (cases) were included from the date of first registration in DiaBase between 2013 and 2018 (index date) and matched 1:5 by year of birth and gender (controls). Controls were assigned the same index date as their matching cases and subsequently excluded if they were registered with a diabetes International Classifications of Disease (ICD)-10 code (E10 or E11) and/or Anatomical Therapeutic Chemical Classification (ATC) = A10A* or A10B*. This exclusion was performed after the 1:5 matching to exclude the diagnosis of diabetes in the control group. Persons with missing data in exposure, outcome or selected confounders were not included in this study, thus we only used a complete data set.

Patients with migraine were identified in NPR through ICD codes for migraine (G43) and treatment of migraine (BAHY2). The DNPR was searched for ATC codes for treatment of migraine, including triptans (N02CC), pizotifen (MN02CX08), and galcanezumab (MN02CX08) with inclusion if the presence of more than one redeemed prescription within a year. Data were linked on person-level from the registers used in this study (NPR, CPR, ITC/ATC). Linked data from the various registers was performed by using the pseudonymized civil registration number (CPR), which is uniquely identifiable, and which is assigned to all citizens in Denmark. This minimizes the risk of bias associated with linkage of data.

The primary endpoint was 5-year incident migraine measured with adjusted hazard ratio (HR) by level of DR at baseline. The secondary endpoint was migraine at index date measured with adjusted odds ratio (OR) by level of DR.

The selection of possible confounders was based on a priori decision. We included data regarding age at first screening for DR, gender, type and duration of diabetes, marital status, Charlson Comorbidity Index (CCI) score^26^, and use of specific medication (insulin, other glucose lowering drugs, and cholesterol lowering drugs). The definition of types of diabetes and codes can be found in supplementary Table S1.

### Statistical analyses

Continuous variables are presented as median with interquartile range (IQR) and categorical variables as frequencies and percentages. We tested for trend with Cuzick's extension of the Wilcoxon rank-sum test for all groups (Table 1). Odds ratios (OR) with 95% confidence interval (CI) for migraine in the cross-sectional study (Tables 3, 4 and Supplementary Table S3) were estimated in crude, age and gender adjusted, and multivariable logistic regression models. Cox proportional hazards regression was used to estimate hazard ratios (HR) with 95% CI for the association between DR and migraine in the prospective study (Tables 3 and 4) in a crude, age and gender adjusted, and a multivariable model. In Tables 2 and 4, we used patients with level 0 as the control group to compare with all other levels of DR. We performed these sub-analyses to eliminate diabetes as the cause of lower prevalence and incidence of migraine and focus on the association between migraine and DR alone. For the analyses, patients with diabetes were stratified according to the level of DR. Cases and controls were both excluded from the prospective study if they were diagnosed with migraine before the index date. We also performed a sub analysis on both HR and OR in the part of the study cohort younger than 55 years, as prevalence of migraine is known to decline above this age^27^. We performed this analysis in both cases and controls to examine, if an assumed higher prevalence of migraine in this younger part of the study cohort reveals additional differences between cases and controls. prevalence of migraine would increase in a younger population.

**Table 1:** Prevalent migraine in patients with diabetes according to level of diabetic retinopathy and their corresponding age- and gender-matched controls.

Level of DR	Patients with diabetes			Age- and gender matched controls			OR (95% CI)		
	Patients with migraine	Total number of patients	Prevalence of migraine (%)	Patients with migraine	Total number of patients	Prevalence of migraine (%)	Crude	Age and gender	Multivariable
All	12,512	205,970	6.1	65,224	1,003,170	6.5	0.93 (0.91;0.95)	0.93 (0.91;0.95)	0.83 (0.81;0.85)
0	10,914	171,795	6.3	54,975	836,865	6.6	0.96 (0.95;0.99)	0.97 (0.94;0.99)	0.87 (0.85;0.89)
1–4	1598	34,175	4.7	10,249	166,305	6.2	0.75 (0.71;0.79)	0.74 (0.70;0.79)	0.67 (0.63;0.71)
1	1045	21,131	4.9	6342	102,788	6.2	0.79 (0.74;0.85)	0.79 (0.73;0.84)	0.70 (0.65;0.76)
2	279	6594	4.2	1853	32,095	5.8	0.72 (0.63;0.82)	0.72 (0.63;0.82)	0.68 (0.59;0.78)
3	44	1162	3.8	350	5681	6.2	0.60 (0.44;0.83)	0.59 (0.43;0.82)	0.51 (0.35;0.73)
4	230	5288	4.3	1704	25,741	6.6	0.64 (0.56;0.74)	0.64 (0.55;0.73)	0.54 (0.46;0.64)

Odds ratio (OR) with 95% confidence interval (CI) for prevalent migraine for patients with diabetes mellitus screened for diabetic retinopathy (DR) (cases) compared to age- and gender-matched controls (1:5) according to level of DR for cases at the time of the first registration in the Danish Registry of Diabetic Retinopathy for cases.

*CI* confidence interval.

Level of DR given by the worse eye.

Multivariable model adjusted for sex, age, marital status, use of lipid lowering drugs and Charlson comorbidity index: myocardial infarct, congestive heart failure, cerebrovascular disease, chronic pulmonary disease, connective tissue disease/rheumatologic disease, ulcer disease, mild liver disease, hemiplegia/hemiplegia or paraplegia, any malignancy (including leukemia and lymphoma), moderate-severe liver disease, solid metastatic tumor, and acquired immunodeficiency syndrome.

P-values below 0.05 and CI’s that did not include 1.0 were considered statistically significant. All statistics were performed using Stata version 16.1 (StataCorp LLC, College Station, TX, USA). All authors had access to the database population in this study.

### Ethics

This project was approved by The Danish Data Protection Agency before conduction. All data applied were pseudo-anonymized. All methods were carried out in accordance with The Danish Data Protection Agency guidelines and regulations.

## Results

**Figure 1:**
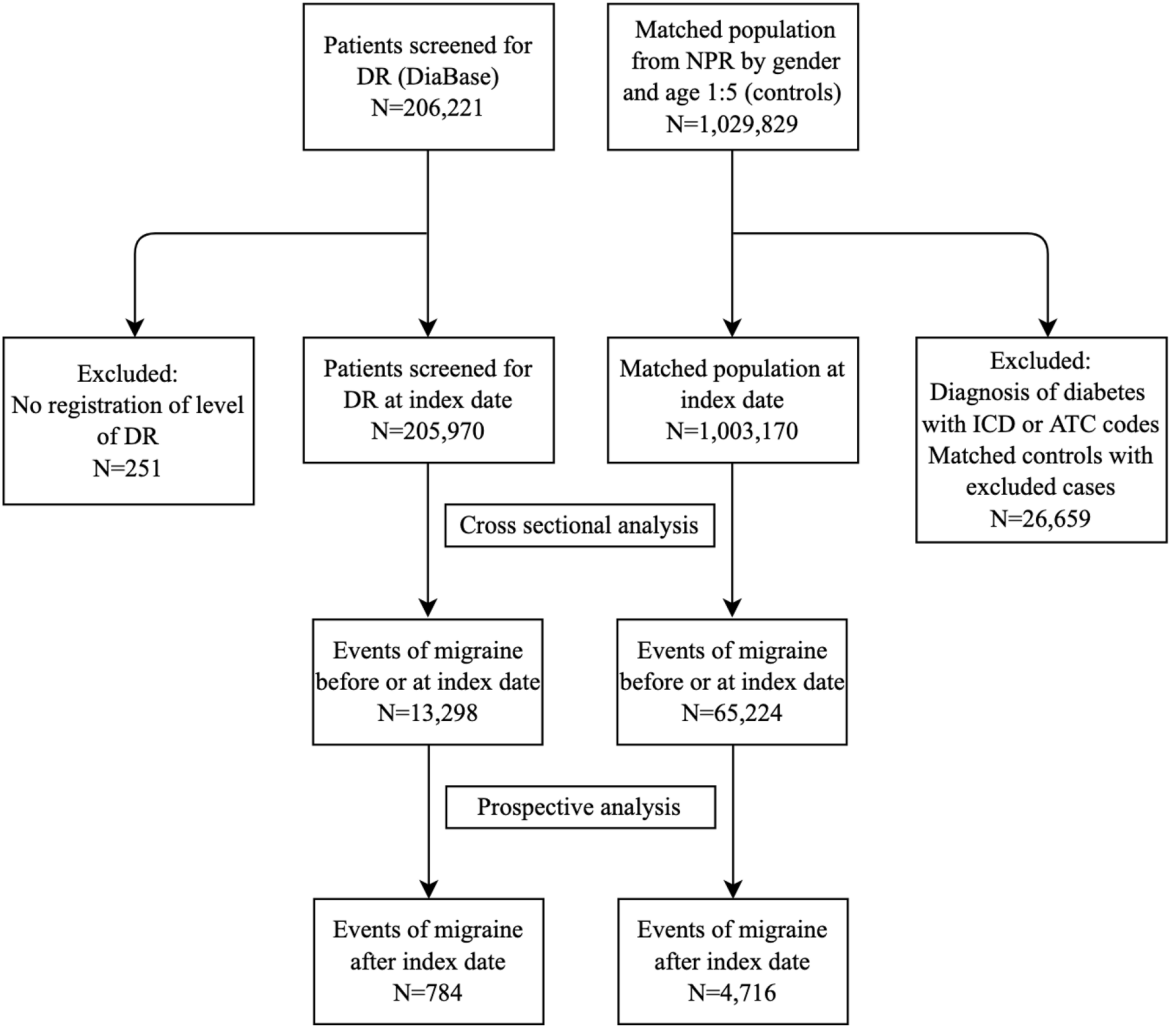
Flow chart of inclusion and exclusion. Flowchart showing patient progression in the study. Diabase: Danish Registry of Diabetic Retinopathy; DR: Diabetic Retinopathy; CPR: The Danish Civil Registration System; ICD: International Classification of Disease; ATC: Anatomical Therapeutic Chemical Classification System.

In this study, we included 205,970 cases from the DiaBase and 1,003,170 matched controls without a diabetes diagnose and no prescriptions of antidiabetic drugs. A flow chart showed in- and exclusion of the study population is presented in Fig. 1.

Characteristics of patients with diabetes are shown in Supplementary Table S2. The study population, who participated in the Danish eye screening program for DR, were more likely to be men, they were more likely diagnosed with type 2 diabetes, married, and having a lower score on CCI. Patients registered with migraine were more likely to be registered with DR level 0, and median age decreased with increasing levels of DR. Patients with type 1 diabetes had a higher level of DR compared to patients with type 2 diabetes and the median duration of diabetes was highest for patients with level 4 DR and type 1 diabetes (20.5 ± 2.7 years). CCI score 1–3 was more likely in patients with level 4 DR, and use of insulin increased with higher levels of DR. The opposite was present for glucose lowering treatment, excl. insulins. The use of antihypertensive drugs was similar for levels 0–3 DR (74.1–76.9%) but higher in level 4 DR (86.0%). The prevalence of migraine was lower in patients with higher levels of DR.

The OR and prevalence for migraine for patients with diabetes in the DiaBase according to the level of DR in comparison with controls are eluded in Table 1. We found a lower prevalence of 17% lower prevalence of migraine in patients with diabetes (OR (95% CI): 0.83 (0.81;0.85)) compared to age- and gender matched controls without diabetes in the multivariable model with prevalence increasing with increasing levels of DR. The same results were present when dividing patients with diabetes into type 1 and type 2 diabetes (Supplementary Tables S3 and S4). We found an interaction between age and DR at index date and therefore present results stratified by age in Supplementary Table S5. When using patients with DR level 0 as the reference group (Table 2), we observed a lower presence of migraine overall (OR (95% CI): 0.69 (0.65–0.72)) and for all levels (1–4) of DR individually compared to level 0.

**Table 2:** Prevalent migraine in patients with diabetes according to level of diabetic retinopathy.

Level of DR	Patients with migraine	Patients without migraine	OR (95% CI)		
			Crude	Age and gender	Multivariable
Level 0	10,914	160,881	1 (reference)	1 (reference)	1 (reference)
Level 1 to 4	1598	32,580	0.72 (0.69–0.76)	0.69 (0.65–0.73)	0.69 (0.65–0.72)
Level 1	1045	20,086	0.77 (0.72–0.82)	0.73 (0.68–0.78)	0.72 (0.68–0.78)
Level 2	279	6315	0.81 (0.76–0.86)	0.81 (0.76–0.86)	0.80 (0.75–0.85)
Level 3	44	1118	0.83 (0.75–0.92)	0.81 (0.73–0.90)	0.80 (0.72–0.89)
Level 4	230	5058	0.90 (0.88–0.94)	0.89 (0.86–0.92)	0.89 (0.86–0.92)

Odds ratio (OR) with 95% confidence interval (CI) for prevalent migraine for patients with diabetes mellitus screened for diabetic retinopathy (DR) at the time of the index date according to level of DR (level 0 used as reference).

*CI* confidence interval.

Level of DR given by the worse eye.

Multivariable model adjusted for sex, age, marital status, use of lipid lowering drugs and Charlson comorbidity index: myocardial infarct, congestive heart failure, cerebrovascular disease, chronic pulmonary disease, connective tissue disease/rheumatologic disease, ulcer disease, mild liver disease, hemiplegia/hemiplegia or paraplegia, any malignancy (including leukemia and lymphoma), moderate-severe liver disease, solid metastatic tumor, and acquired immunodeficiency syndrome.

The HR for developing migraine for patients screened for DR by level of DR and controls are displayed in Table 3. Patients with diabetes (cases) had a lower risk of developing migraine (level 1–4 HR (95% CI): 0.66 (0.55–0.80)) compared to controls (Table 3). This was especially apparent for DR level 4 in the multivariable model (HR: 0.53 95% CI: 0.32–0.88). Furthermore, this risk was especially low in patients with type 1 diabetes (level 1–4 HR (95% CI): 0.55 (0.42–0.73)) (Supplementary Table S6). When using patients with DR level 0 as the reference group, there were no statistically significant differences in risk of migraine in any of the groups (Table 4). This was also apparent when dividing patients into type 1 and type 2 diabetes, except for the crude model in type 2 diabetes, where there was a 33% less risk of developing migraine in the 5-year prospective study (level 0 vs. level 1–4 HR (95% CI): 0.67 (0.47–0.97)) (Supplementary Table S7).

**Table 3:** Incident migraine in patients with diabetes according to level of diabetic retinopathy and their corresponding age- and gender-matched controls.

Level of DR	Patients with diabetes		Age- and gender matched controls		HR (95% CI)		
	Events	Person-years	Events	Person-years	Crude	Age and gender	Multivariable
All	784	623,261	4716	3,015,920	0.80 (0.75;0.87)	0.81 (0.75;0.87)	0.76 (0.70;0.82)
0	617	501,244	3619	2,419,531	0.82 (0.76;0.90)	0.83 (0.76;0.90)	0.79 (0.71;0.87)
1–4	167	122,016	1097	596,389	0.74 (0.63;0.88)	0.74 (0.63;0.87)	0.66 (0.55;0.80)
1	99	74,975	655	363,764	0.73 (0.59;0.90)	0.73 (0.59;0.90)	0.67 (0.53;0.85)
2	35	23,490	215	115,471	0.80 (0.56;1.14)	0.78 (0.55;1.12)	0.66 (0.44;0.99)
3	10	4056	41	20,116	1.20 (0.61;2.41)	1.18 (0.59;2.36)	1.20 (0.54;2.64)
4	23	19,494	186	97,038	0.61 (0.40;0.95)	0.60 (0.39;0.92)	0.53 (0.32–0.88)

Hazard ratio (HR) with 95% confidence interval (CI) for 5-year incident migraine after index date for patients with diabetes mellitus screened for diabetic retinopathy (DR) and age- and gender-matched controls according to level of DR for cases.

*CI* confidence interval.

Level of DR given by the worse eye.

Multivariable model adjusted adjusted for sex, age, marital status, use of lipid lowering drugs and Charlson comorbidity index: myocardial infarct, congestive heart failure, cerebrovascular disease, chronic pulmonary disease, connective tissue disease/rheumatologic disease, ulcer disease, mild liver disease, hemiplegia/hemiplegia or paraplegia, any malignancy (including leukemia and lymphoma), moderate-severe liver disease, solid metastatic tumor, and acquired immunodeficiency syndrome.

**Table 4:** Incident migraine in patients with diabetes according to level of diabetic retinopathy.

Level of DR	Events of migraine	Person-years	HR (95% CI)		
			Crude	Age and gender	Multivariable
Level 0	617	501,244	1 (reference)	1 (reference)	1 (reference)
Level 1 to 4	167	122,016	1.12 (0.94–1.33)	0.94 (0.80–1.12)	0.93 (0.78–1.11)
Level 1	99	74,975	1.08 (0.87–1.33)	0.89 (0.72–1.10)	0.88 (0.71–1.10)
Level 2	35	23,490	1.22 (0.86–1.71)	1.13 (0.80–1.58)	1.08 (0.76–1.53)
Level 3	10	4056	2.01 (1.08–3.75)	1.49 (0.80–2.79)	1.46 (0.78–2.74)
Level 4	23	19,494	0.96 (0.64–1.46)	0.86 (0.57–1.30)	0.83 (0.55–1.27)

Hazard ratio (HR) with 95% confidence interval (CI) for 5-year incident migraine after the index date for patients with diabetes mellitus screened for diabetic retinopathy (DR) according to level of DR (level 0 used as reference).

*CI* confidence interval.

Level of DR given by the worse eye.

Multivariable model adjusted adjusted for sex, age, marital status, use of lipid lowering drugs and Charlson comorbidity index: myocardial infarct, congestive heart failure, cerebrovascular disease, chronic pulmonary disease, connective tissue disease/rheumatologic disease, ulcer disease, mild liver disease, hemiplegia/hemiplegia or paraplegia, any malignancy (including leukemia and lymphoma), moderate-severe liver disease, solid metastatic tumor, and acquired immunodeficiency syndrome.

Sub analyses on both HR and OR on the population (also divided into type 1 and type 2 diabetes) where we excluded patients older than 55 years did not alter the above-mentioned results (results not shown).

## Discussion

In this study, we found a lower prevalence of migraine amongst patients with diabetes within all levels of DR (including level 0) compared to age- and gender matched controls without diabetes. We also found a lower incidence of migraine among patients with diabetes, but this did not depend upon the presence of DR, suggesting that diabetes may act as a protecting marker of migraine.

This is partly in line with previous findings of a lower prevalence of migraine in patients with diabetes compared to matched controls without^7–9^. However, these studies did not explore the relation with DR which this study adds new insights into. Though the complete pathophysiology is not known in detail, a proposed mechanism behind migraine includes a combination of vasogenic and neurogenic factors, including vasodilation and overactivation of nerves^10,28^. It could be hypothesized that arteriosclerosis of vessels present in patients with diabetes and DR^15^, leads to increased stiffness^29^ with reduced ability to dilate the vessels^30^, which in turn will cause an inadequate response to impulses in brain vessels associated with migraine. Furthermore, DR also possesses neurodegenerative aspects, which could, furthermore, lead to a weaker response^31–33^. Activation of glial cells via cellular pathways have shown to increase the pain-response in migraine^34^. Müller cells are thought to be the principal glial cell of the retina, and dysfunction of these cells has been proposed to play an important part in the development of DR^35^. Furthermore, hyperglycemia have shown to cause apoptosis of retinal Müller cells in a rat model^36^. However hypothetical, dysfunction and apoptosis of these cells may represent similar mechanisms present in glial cells in migraine causing decreased pain response. The degree of DR could perhaps act as a predictor of the development of migraine with more severe stages being protective because of the aforementioned pathophysiological mechanisms. However, as the results presents, it is not quite clear yet whether diabetes or the degree of DR is the main cause for a lower prevalence of migraine.

Calcitonin gene-related peptide (CGRP) is thought to play an important role in inducing the response occurring during a migraine attack by its vasodilating effect^37^. In rats, it has been shown that the apoptosis of retinal cells that is present in DR was confined to the ganglion cell layer. CGRP is normally located in the ganglion cell layer^38^ of the retina. As this layer is often affected by apoptosis in DR, it is possible the production of CGRP may decline. It can be speculated whether this mechanism is also present in other cells in the body, with the general metabolic stress that is present in diabetes^39^. This could possibly reduce the production of CGRP and therefore be protective against migraine attacks, as CGRP normally causes vasodilation.

When applying level 0 DR as the reference group the prevalence of migraine was lower overall (31%) making the association between DR and migraine evident (Table 2). This implies that diabetes is not the sole cause of less prevalent migraine, as other aspects such as HbA1c could also play a role. In the prospective study, where level 1–4 DR was compared to level 0, there were no associations between migraine and DR.

A previous study by Hagen et al. only found an inverse relationship between prevalent migraine in patients with type 1 diabetes but not in those with type 2 diabetes^7^. In our study we found that prevalent migraine was lower in both type 1 and type 2 diabetes. Although the age of patients in Hagen’s study population was similar to ours, the sample size was much smaller. Our results are furthermore supported by another previous study by Berge et al. that found lower prevalent migraine in both persons using insulin alone and persons using non-insulin diabetes medication only; however, this was only present in patients from the age of 50^8^. Conversely, our study found that this was present in patients with diabetes both above and under 55 years of age in incident migraine. Berge et al. only used prescriptions of medication to categorize patients, where our study participants were categorized by a clinical diagnosis of type of diabetes. Both types of categorizations of diabetes offer uncertainties, and it is difficult to conclude why results are conflicting. We did not find any differences between prevalent migraine when dividing patients into type 1 and type 2 diabetes, although these results should be analyzed with caution, because of the low number of patients.

The prevalence of migraine is presumably underestimated, both because of under-diagnosis by health care staff, but also because patients do not always seek professional health care for their symptoms and self-medicate instead^1^. This underestimation could very well be present in this study as well, as we relied on the registered prescription of migraine medication. It cannot be excluded that there are patients that suffer from migraine but sufficiently manage to self-medicate with non-prescription medicine. These would not be accounted for in this study, which could have caused an underrepresentation of patients with a mild degree of migraine. This underestimation would most likely be present among controls, as patients with diabetes are regularly in contact with health care systems and may have a higher probability to be treated for migraine.

Limitations of this study included that we did not have data of hemoglobin A1c (HbA1c) and racial background. Likewise, migraine is in particular present in younger persons^40^, and given that the average age for our cohort was 65 years, migraine may be underrepresented compared to the general population. Furthermore, a course of 5 years may not be enough to follow a development in migraine. Another possible cause of underestimation of migraine in the diabetes population of this study could be the use of angiotensin-converting enzyme inhibitors which in some studies has been suggested to work beneficial in reducing migraine symptoms^41^. Another possible limitation involves the fact that our data did not include information about body mass index (BMI), as obesity have previously been associated with migraine with increased prevalence and increased number of attacks in both a Norwegian and Chinese population^42,43^. Finally, our study may not have been powered to associate migraine with higher levels of DR, as we had a relatively low number of those patients. Misclassification bias should always be taken into consideration when completing a register-based study. Patients in the control group could potentially have diabetes without this being registered and this may be a limitation as well.

Strengths of this study included the large sample size with the inclusion of a full national cohort of patients attending the national eye screening program for DR. Furthermore, access to well-established databases and national registers gave us relevant and important information about the level of DR comorbidities and medication use.

Conclusively, in a national study of more than 1.2 million people, patients screened for DR had a lower risk of present migraine. However, we could not demonstrate that the presence and degree of DR was a biomarker of protection of incident migraine. These results suggest that DR and especially diabetes, in combination, acts as a protector against developing migraine. Furthermore, it provides important insight into the degree to which DR is associated with other components of the body, in this instance the brain. These results provide new insights into the complex aspects of the pathophysiological mechanisms in diabetes and DR which provide further knowledge in the understanding of these diseases.

## Supplementary Information

Supplementary Tables.

## Data Availability

Protocol, raw data, and computing code required to replicate the results in our report can be obtained by contacting corresponding author upon reasonable request.

## Abbreviations

ATC: Anatomical Therapeutic Chemical Classification
CCI: Charlson Comorbidity Index
CGRP: Calcitonin gene-related peptide
Diabase: The Danish National Registry of Diabetic Retinopathy
DNPR: Danish National Prescription Registry
DR: Diabetic retinopathy
ICD: International Classifications of Disease
NPR: National Patient Register
