# Supplementary material for: Investigation of the correlation between diabetic retinopathy and prevalent and incident migraine in a national cohort study
Source: Sci Rep. 2022 Jul 20;12:12443. doi: 10.1038/s41598-022-16793-0 (PMC9300720; doi:10.1038/s41598-022-16793-0)
Supplement: Supplementary file 1 — Supplementary Tables. [file 41598_2022_16793_MOESM1_ESM.docx]

**Supplementary Table S1**

Type of diabetes determined according to International Classification of Diseases (ICD) version 10 codes for diabetes and Anatomical Therapeutic Chemical Classification (ATC) codes for treatment of diabetes given at the time of the first entry in the Danish Registry of Diabetic Retinopathy.

| ICD version 10 code for diabetes* | | ATC code for diabetes treatment^†^ | | Type of diabetes |
| --- | --- | --- | --- | --- |
| DE10* | DE11* | A10A* | A10B* |  |
| Yes | No | No | No | Unknown |
| Yes | Yes | No | No | Unknown |
| Yes | No | Yes | No | Type 1 diabetes |
| Yes | No | No | Yes | Unknown |
| Yes | No | Yes | Yes | Type 1 diabetes |
| Yes | Yes | No | Yes | Unknown |
| Yes | Yes | Yes | No | Unknown |
| Yes | Yes | Yes | Yes | Unknown |
| No | Yes | No | No | Type 2 diabetes |
| No | No | Yes | No | Unknown |
| No | No | No | Yes | Type 2 diabetes |
| No | Yes | Yes | No | Type 2 diabetes |
| No | Yes | No | Yes | Type 2 diabetes |
| No | No | Yes | Yes | Type 2 diabetes |
| No | Yes | Yes | Yes | Type 2 diabetes |
| No | No | No | No | Type 2 diabetes |

*ICD codes: DE10*: type 1 diabetes, DE11* type 2 diabetes. ^†^ATC codes for redeemed prescriptions: A10A*: insulin, A10B*: blood glucose lowering drugs excl. insulin.

**Supplementary Table S2**

Characteristics of patients with diabetes mellitus in the Danish Registry of Diabetic Retinopathy according to the level of diabetic retinopathy (DR).

|  | **Overall** | **Level of DR** | | | | |  |
| --- | --- | --- | --- | --- | --- | --- | --- |
|  |  | **0** | **1** | **2** | **3** | **4** | **P value** |
| **Number of patients, n** | 205,970 | 171,795 | 21,131 | 6,594 | 1,162 | 5,288 |  |
| **Gender, n(%) male** | 116,534 (56.6) | 95,843 (55.8) | 12,522 (59.3) | 4,159 (63.1) | 787  (67.7) | 3,223 (60.9) | <0.001 |
| **Age, years (IQR)** | 65.7 (55.4;73.1) | 66.2 (56.3;73.4) | 63.5 (51.5;72.2) | 62.0 (51.4;70.7) | 55.9 (45.6;66.0) | 61.3 (50.3;70.3) | <0.001 |
| **Type of diabetes, n (%)** |  |  |  |  |  |  | <0.001 |
| **Type 1 diabetes** | 16,999 (11.5) | 9,490 (8.2) | 4,571 (23.5) | 1,132 (18.0) | 228  (20.2) | 1,578 (30.3) |  |
| **Type 2 diabetes** | 95,720 (64.5) | 84,293 (72.4) | 7,784 (40.1) | 2,392 (37.9) | 387  (34.2) | 864  (16.6) |  |
| **Unknown** | 35,733 (24.1) | 22,605 (19.4) | 7,065 (36.4) | 2,780 (44.1) | 515  (45.6) | 2,768 (53.1) |  |
| **Duration of diabetes, years (IQR)** |  |  |  |  |  |  | <0.001 |
| **Type 1 diabetes** | 16.7 (7.4;20.4) | 9.7 (3.6;18.7) | 19.6 (15.1;21.0) | 19.7 (16.4;21.3) | 19.5 (16.8;20.7) | 20.5 (19.5;22.2) |  |
| **Type 2 diabetes** | 6.9 (3.2;11.5) | 6.5 (3.0;10.7) | 11.6 (6.8;16.1) | 11.6 (6.5;16.2) | 12.1 (6.4;16.0) | 14.8 (9.2;19.1) |  |
| **Unknown** | 13.9 (8.4;19.2) | 11.3 (6.4;16.3) | 17.7 (13.2;20.0) | 17.5 (12.9;20.0) | 17.1 (13.0;19.7) | 19.8 (18.1;21.2) |  |
| **Marital status, n (%)** |  |  |  |  |  |  | <0.001 |
| **Never married** | 30,904 (15.0) | 24,524 (14.3) | 3,775 (17.9) | 1,247 (18.9) | 283  (24.4) | 1075 (20.3) |  |
| **Married** | 118,764 (57.7) | 99,847 (58.1) | 11,820 (55.9) | 3,634 (55.1) | 588  (50.6) | 2875 (54.4) |  |
| **Widowed or**  **divorced** | 56,302 (27.3) | 47,424 (27.6) | 5,536 (26.2) | 1,713 (26.0) | 291  (25.0) | 1,338 (25.3) |  |
| **Charlson Comorbidity Index score, n (%)** |  |  |  |  |  |  | <0.001 |
| **0 (low)** | 148,615 (72.2) | 129,907 (75.6) | 12,730 (60.2) | 3,555 (53.9) | 576  (49.6) | 1,847 (34.9) |  |
| **1 (moderate low)** | 27,728 (13.5) | 18,248 (10.6) | 5,010 (23.7) | 1,892 (28.7) | 387  (33.3) | 2,191 (41.4) |  |
| **2 (moderate high)** | 18,721 (9.1) | 15,436 (9.0) | 1,852  (8.8) | 615  (9.3) | 114  (9.8) | 704  (13.3) |  |
| **≥3 (high)** | 10,906 (5.3) | 8,204  (4.8) | 1,539  (7.3) | 532  (8.1) | 85  (7.3) | 546  (10.3) |  |
| **Use of medication, n (%)** |  |  |  |  |  |  |  |
| **Insulin** | 8,000 (3.9) | 6,766 (3.9) | 756 (3.6) | 327 (5.0) | 67 (5.8) | 84 (1.6) | <0.001 |
| **Glucose lowering treatment, excl. insulins** | 23,634 (11.5) | 22,392 (13.0) | 807 (3.8) | 303 (4.6) | 66 (5.7) | 66 (1.2) | <0.001 |
| **Antihypertensive drugs** | 6,344 (3.1) | 5,421 (3.2) | 505 (2.4) | 245 (3.7) | 73 (6.3) | 100 (1.9) | <0.001 |
| **Cholesterol lowering drugs** | 10,902 (5.3) | 9,775 (5.7) | 668 (3.2) | 279 (4.2) | 70 (6.0) | 110 (2.1) | <0.001 |
| **Migraine, n (%)** | 13,298 (6.5) | 11,533 (6.7) | 1,144 (5.4) | 314 (4.8) | 54 (4.6) | 253 (4.8) | <0.001 |

Level of DR given by the worse eye.

**Supplementary Table S3 – prevalent migraine in patients with diabetes according to level of diabetic retinopathy and type 1 diabetes and their corresponding age- and gender-matched controls**

Odds ratio (OR) with 95% confidence interval (CI) for prevalent migraine for patients with diabetes mellitus screened for diabetic retinopathy (DR) (cases) compared to age- and gender-matched controls (1:5) according to level of DR for cases at the time of the first registration in the Danish Registry of Diabetic Retinopathy for cases.

| Level of DR | Patients with type 1 diabetes | | | Age- and gender matched controls | | | OR (95% CI) | | |
| --- | --- | --- | --- | --- | --- | --- | --- | --- | --- |
|  | Patients with migraine | Total number of patients | Prevalence of migraine | Patients with migraine | Total number of patients | Prevalence of migraine | Crude | Age and gender | Multivariable |
| All | 913 | 16,999 | 6.3% | 5,598 | 83,391 | 6.7% | 0.79 (0.73;0.85) | 0.78 (0.73;0.84) | 0.69 (0.64;0.75) |
| 0 | 495 | 9,490 | 6.1% | 3,021 | 46,661 | 6.5% | 0.79 (0.72;0.88) | 0.79 (0.72;0.87) | 0.71 (0.64;0.79) |
| 1-4 | 418 | 7,091 | 5.9% | 2,577 | 34,153 | 7.5% | 0.78 (0.70;0.87) | 0.78 (0.70;0.86) | 0.66 (0.58;0.74) |
| 1 | 273 | 4,571 | 6.8% | 1,576 | 22,368 | 7.0% | 0.84 (0.73;0.96) | 0.83 (0.73;0.95) | 0.69 (0.60;0.80) |
| 2 | 55 | 1,132 | 5.7% | 326 | 5,536 | 5.9% | 0.82 (0.61;1.09) | 0.81 (0.61;1.09) | 0.68 (0.49;0.95) |
| 3 | 6 | 98 | 3.9% | 79 | 1,117 | 7.1% | 0.36 (0.15;0.82) | 0.34 (0.15;0.80) | 0.36 (0.14;0.92) |
| 4 | 84 | 1578 | 6.2% | 596 | 7706 | 7.7% | 0.67 (0.53;0.85) | 0.66 (0.52;0.84) | 0.58 (0.44;0.77) |

**Supplementary Table S4 – prevalent migraine in patients with diabetes according to level of diabetic retinopathy and type 2 diabetes and their corresponding age- and gender-matched controls**

Odds ratio (OR) with 95% confidence interval (CI) for prevalent migraine for patients with diabetes mellitus screened for diabetic retinopathy (DR) (cases) compared to age- and gender-matched controls (1:5) according to level of DR for cases at the time of the first registration in the Danish Registry of Diabetic Retinopathy for cases.

| Level of DR | Patients with type 2 diabetes | | | Age- and gender matched controls | | | OR (95% CI) | | |
| --- | --- | --- | --- | --- | --- | --- | --- | --- | --- |
|  | Patients with migraine | Total number of patients | Prevalence of migraine | Patients with migraine | Total number of patients | Prevalence of migraine | Crude | Age and gender | Multivariable |
| All | 9247 | 153,238 | 6.4% | 48,765 | 746,148 | 7.0% | 0.92 (0.90;0.94) | 0.92 (0.90;0.94) | 0.84 (0.81;0.86) |
| 0 | 8745 | 139,700 | 6.7% | 45,030 | 680,307 | 7.1% | 0.94 (0.92;0.96) | 0.94 (0.92;0.97) | 0.86 (0.84;0.88) |
| 1-4 | 502 | 13,036 | 3.9% | 3,735 | 62,106 | 6.0% | 0.64 (0.58;0.70) | 0.64 (0.58;0.70) | 0.58 (0.52;0.65) |
| 1 | 379 | 9,495 | 4.2% | 2,644 | 46,142 | 6.1% | 0.68 (0.61;0.76) | 0.68 (0.61;0.76) | 0.62 (0.55;0.71) |
| 2 | 89 | 2,682 | 3.4% | 707 | 13,059 | 5.7% | 0.60 (0.48;0.75) | 0.60 (0.48;0.75) | 0.59 (0.46;0.76) |
| 3 | 13 | 419 | 3.2% | 125 | 2,054 | 6.5% | 0.49 (0.28;0.88) | 0.49 (0.27;0.88) | 0.32 (0.16;0.62) |
| 4 | 21 | 942 | 2.3% | 259 | 4,586 | 6.0% | 0.38 (0.24;0.60) | 0.38 (0.24;0.59) | 0.30 (0.19;0.49) |

CI= confidence interval.

Level of DR given by the worse eye.

Multivariable model adjusted for sex, age, marital status, use of lipid lowering drugs and Charlson comorbidity index: myocardial infarct, congestive heart failure, cerebrovascular disease, chronic pulmonary disease, connective tissue disease / rheumatologic disease, ulcer disease, mild liver disease, hemiplegia/ hemiplegia or paraplegia, any malignancy (including leukemia and lymphoma), moderate-severe liver disease, solid metastatic tumor, and acquired immunodeficiency syndrome.

Please note that patients without diabetes was matched 1:5 in each level of DR why they are presented under each level.

**Supplementary Table S5**

Odds ratio (OR) for migraine for patients with diabetes mellitus in the Danish Registry of Diabetic Retinopathy according to level of diabetic retinopathy (DR).

| Level of DR | Patients with diabetes | | | Patients without diabetes | | | OR (95% CI) | | |
| --- | --- | --- | --- | --- | --- | --- | --- | --- | --- |
|  | Patients with migraine | Total number of patients | Prevalence of migraine | Patients with migraine | Total number of patients | Prevalence of migraine | Crude | Age- and gender | Multivariable |
| Below or 65 years of age | | | | | | | | | |
| All | 8,252 | 98,639 | 8.4% | 39,543 | 482,997 | 8.2% | 1.02 (0.99;1.0) | 1.02 (1.0;1.05) | 0.89 (0.86;0.92) |
| 0 | 7,072 | 79,475 | 8.9% | 32,554 | 389,384 | 8.4% | 1.07 (1.04;1.10) | 1.07 (1.04;1.20) | 0.93 (0.90;0.96) |
| 1-4 | 1,180 | 19,164 | 6.2% | 6,989 | 93,613 | 7.5% | 0.81 (0.76;0.97) | 0.81 (0.76;0.86) | 0.70 (0.90;0.96) |
| 1 | 760 | 11,338 | 6.7% | 4,185 | 55,463 | 7.5% | 0.88 (0.81;0.95) | 0.88 (0.81;0.95) | 0.75 (0.68;0.83) |
| 2 | 207 | 3,841 | 5.4% | 1,268 | 18,765 | 6.8% | 0.79 (0.68;0.91) | 0.78 (0.87;0.91) | 0.72 (0.60;0.86) |
| 3 | 36 | 836 | 4.3% | 282 | 4,094 | 6.9% | 0.61 (0.43;0.87) | 0.60 (0.42;0.86) | 0.57 (0.37;0.87) |
| 4 | 177 | 3,149 | 5.6% | 1,254 | 15,291 | 8.2% | 0.67 (0.57;0.78) | 0.66 (0.56;0.78) | 0.55 (0.45;0.70) |
| Above 65 years of age | | | | | | | | | |
| All | 4,260 | 107,331 | 3.9% | 25,681 | 520,173 | 4.9% | 0.80 (0.77;0.82) | 0.79 (0.77;0.82) | 0.76 (0.73;0.79) |
| 0 | 3,842 | 92,320 | 4.2% | 22,421 | 447,481 | 5.0% | 0.82 (0.79;0.85) | 0.82 (0.79;0.85) | 0.78 (0.76;0.82) |
| 1-4 | 418 | 15,011 | 2.9% | 3,260 | 72,692 | 4.5% | 0.61 (0.55;0.68) | 0.61 (0.55;0.68) | 0.58 (0.52;0.65) |
| 1 | 285 | 9,793 | 2.9% | 2,157 | 47,325 | 4.6% | 0.63 (0.55;0.71) | 0.63 (0.55;0.71) | 0.60 (0.52;0.69) |
| 2 | 72 | 2,753 | 2.6% | 585 | 13,330 | 4.4% | 0.59 (0.46;0.75) | 0.58 (0.46;0.75) | 0.58 (0.45;0.77) |
| 3 | 8 | 326 | 2.5% | 68 | 1,587 | 4.3% | 0.56 (0.27;1.18) | 0.56 (0.26;1.18) | 0.35 (0.15;0.82) |
| 4 | 53 | 2,139 | 2.5% | 450 | 10,450 | 4.3% | 0.56 (0.42;0.75) | 0.56 (0.42;0.75) | 0.51 (0.37;0.69) |

Level of DR given by the worse eye.

Multivariable model adjusted adjusted for sex, age, marital status, use of lipid lowering drugs and Charlson comorbidity index: myocardial infarct, congestive heart failure, cerebrovascular disease, chronic pulmonary disease, connective tissue disease / rheumatologic disease, ulcer disease, mild liver disease, hemiplegia/ hemiplegia or paraplegia, any malignancy (including leukemia and lymphoma), moderate-severe liver disease, solid metastatic tumor, and acquired immunodeficiency syndrome.

**Supplementary Table S6 - incident migraine in patients with type 1 and type 2 diabetes according to level of diabetic retinopathy and their corresponding age- and gender-matched controls in 5 years**

Hazard ratio (HR) with 95% confidence interval (CI) for 5-year incident migraine after index date for patients with diabetes mellitus (divided into type 1 and type 2 diabetes) screened for diabetic retinopathy (DR) and age- and gender-matched controls according to level of DR for cases.

| Level of DR | Patients with type 1 diabetes | | Patients without diabetes | | HR (95% CI) | | |
| --- | --- | --- | --- | --- | --- | --- | --- |
|  | Events | Person-years | Events | Person-years | Crude | Age and gender | Multivariable |
| All | 150 | 65,190 | 1135 | 312,202 | 0.63 (0.53;0.75) | 0.63 (0.53;0.75) | 0.54 (0.45;0.65) |
| 0 | 83 | 34,191 | 634 | 164,187 | 0.63 (0.50;0.79) | 0.62 (0.50;0.78) | 0.53 (0.42;0.67) |
| 1-4 | 67 | 30,998 | 501 | 148,014 | 0.64 (0.49;0.82) | 0.64 (0.49;0.82) | 0.55 (0.42;0.73) |
|  | Patients with type 2 diabetes | | Patients without diabetes | | HR (95% CI) | | |
|  | Events | Person-years | Events | Person-years | Crude | Age and gender | Multivariable |
| All | 451 | 437,665 | 2601 | 2,093,152 | 0.83 (0.75;0.92) | 0.84 (0.76;0.93) | 0.82 (0.73;0.92) |
| 0 | 420 | 394,272 | 2378 | 1,885,896 | 0.85 (0.76;0.94) | 0.86 (0.78;0.95) | 0.84 (0.75;0.95) |
| 1-4 | 31 | 43,392 | 223 | 207,255 | 0.66 (0.46;0.97) | 0.66 (0.46;0.97) | 0.56 (0.37;0.87) |

CI= confidence interval.

Level of DR given by the worse eye.

Multivariable model adjusted adjusted for sex, age, marital status, use of lipid lowering drugs and Charlson comorbidity index: myocardial infarct, congestive heart failure, cerebrovascular disease, chronic pulmonary disease, connective tissue disease / rheumatologic disease, ulcer disease, mild liver disease, hemiplegia/ hemiplegia or paraplegia, any malignancy (including leukemia and lymphoma), moderate-severe liver disease, solid metastatic tumor, and acquired immunodeficiency syndrome.

Please note that patients without diabetes was matched 1:5 in each level of DR why they are presented under each level.

**Table S7 - prevalent migraine in patients with diabetic retinopathy**

Odds ratio (OR) with 95% confidence interval (CI) for prevalent migraine for patients with diabetes mellitus (divided into type 1 and type 2 diabetes) screened for diabetic retinopathy (DR) at the time of the index date according to level of DR (level 0 used as reference).

|  | Patients with type 1 diabetes | | OR (95% CI) | | |
| --- | --- | --- | --- | --- | --- |
| Level of DR | Patients with migraine | Patients without migraine | Crude | Age and gender | Multivariable |
| Level 0 | 83 | 34,191 | 1 (reference) | 1 (reference) | 1 (reference) |
| Level 1 to 4 | 67 | 30,998 | 0.90 (0.65;1.24) | 1.26 (0.90;1.77) | 1.22 (0.87;1.72) |
|  | Patients with type 2 diabetes | | OR (95% CI) | | |
| Level of DR | Patients with migraine | Patients without migraine | Crude | Age and gender | Multivariable |
| Level 0 | 420 | 394,272 | 1 (reference) | 1 (reference) | 1 (reference) |
| Level 1 to 4 | 31 | 43,392 | 0.67 (0.47;0.97) | 0.75 (0.52;1.09) | 0.74 (0.51;1.08) |

CI= confidence interval.

Level of DR given by the worse eye.

Multivariable model adjusted adjusted for sex, age, marital status, use of lipid lowering drugs and Charlson comorbidity index: myocardial infarct, congestive heart failure, cerebrovascular disease, chronic pulmonary disease, connective tissue disease / rheumatologic disease, ulcer disease, mild liver disease, hemiplegia/ hemiplegia or paraplegia, any malignancy (including leukemia and lymphoma), moderate-severe liver disease, solid metastatic tumor, and acquired immunodeficiency syndrome.

Please note that patients without diabetes was matched 1:5 in each level of DR why they are presented under each level.
